# Transformation of juvenile idiopathic arthritis in adult-oriented rheumatology care

**DOI:** 10.55730/1300-0144.6098

**Published:** 2025-10-19

**Authors:** Şerife Asya GERME DAĞLIOĞLU, Zeynep BALIK, Zehra ÖZSOY, Yunus Emre DALKILIÇ, Ezgi Deniz BATU, Özge BAŞARAN, Yelda BİLGİNER, Umut KALYONCU, Seza ÖZEN, Şule APRAŞ BİLGEN, Levent KILIÇ

**Affiliations:** 1 Division of Rheumatology, Department of Internal Medicine, Faculty of Medicine, Hacettepe University, Ankara, Turkiye; 2 Division of Rheumatology, Department of Pediatrics, Faculty of Medicine, Hacettepe University, Ankara, Turkiye; 3 Department of Internal Medicine, Faculty of Medicine, Hacettepe University, Ankara, Turkiye

**Keywords:** Classification, juvenile idiopathic arthritis, transitional care, treatment

## Abstract

**Background/aim:**

Juvenile idiopathic arthritis (JIA) is the most prevalent form of chronic inflammatory arthritis in children, and the symptoms persist into adulthood for a considerable number of patients. This study aimed to compare the JIA subtypes using the diagnostic criteria used for adults and identify the clinical characteristics and treatment approaches for patients with JIA transitioning to adult-oriented rheumatology care.

**Materials and methods:**

Patients diagnosed with JIA in the pediatric rheumatology clinic were retrospectively evaluated. The patients (n=107) who had at least one follow-up visit in the adult rheumatology clinic were included in the study. After transitioning from pediatric to adult-oriented rheumatology care, 2 experienced adult rheumatologists retrospectively reclassified the patients based on the adult classification criteria with clinical, serological, and radiological findings.

**Results:**

The most common diagnosis was enthesitis-related arthritis (49.5%), followed by oligoarticular JIA (22.4%). The follow-up diagnoses of the JIA patients in adult-oriented rheumatology care were radiographic axial spondyloarthritis (SpA) (30.8%), nonradiographic axial SpA (15%), rheumatoid arthritis (12.1%), Still’s disease (11.2%), psoriatic arthritis (2.8%), and peripheral SpA (2.8%). However, 25.2% of the patients were unclassified, particularly in the oligoarticular subgroup. During the transition, 60% of the patients with JIA were receiving medical treatment.

**Conclusion:**

A significant number of patients in oligoarticular and rheumatoid factor-negative polyarticular JIA groups did not meet adult classification criteria, making them the most challenging subtypes to manage in adult-oriented rheumatology care. Understanding the transformation of distinct phenotypes into adulthood and managing the transition without interruption can improve the prognosis of JIA.

## 1. Introduction

Juvenile idiopathic arthritis (JIA) is the most commonly encountered chronic inflammatory arthritis among children. The International League of Associations for Rheumatology (ILAR) classifies JIA into 7 subtypes: oligoarticular JIA, enthesitis-related arthritis (ERA), psoriatic arthritis (PsA), undifferentiated arthritis, systemic JIA, rheumatoid factor (RF)-positive polyarticular JIA, and RF-negative polyarticular JIA according to the clinical and laboratory findings within the first 6 months after disease onset [1].

The determination of JIA subtypes relies on clinical symptoms, laboratory findings, family history, and imaging techniques such as ultrasound and magnetic resonance imaging [2].

Therapeutic intervention for JIA typically begins at diagnosis with nonsteroidal anti-inflammatory drugs (NSAIDs), followed by conventional synthetic disease-modifying antirheumatic drugs (csDMARDs), with methotrexate being the most commonly used. Intra-articular steroid injections are effective in managing synovitis and may serve as a first-line treatment for oligoarthritis, either alone or in combination with disease-modifying antirheumatic drugs (DMARDs). Systemic corticosteroids provide effective short-term relief, particularly in systemic JIA (sJIA) patients, but do not impact long-term disease outcomes. The advent of biologic disease-modifying antirheumatic drugs (bDMARDs) has significantly improved patient outcomes, making inactive disease and low disease activity achievable [3,4].

Despite the improvement in treatment options, symptoms persist into adulthood for around 30–60% of JIA patients, requiring continual administration of immunosuppressive treatments [5–7]. Therefore, transitional care is crucial to improve the ongoing care and well-being of patients. There are numerous obstacles regarding the transitional care for JIA patients, hindering its success and effectiveness. There is a lack of standardized criteria for evaluating disease activity in adult JIA patients. While treatment protocols for pediatric JIA are well defined, there is a scarcity of evidence-based guidelines tailored to the unique needs of adult JIA patients. Moreover, lack of follow-up due to remission or transition from pediatric to adult-oriented rheumatology care has led to an insufficient understanding of the adult consequences of JIA [8–12].

Our study aimed to compare the JIA subtypes with the diagnostic criteria used for adults, as well as to identify the clinical characteristics and treatment approaches for patients with JIA transitioning to adult-oriented rheumatology care.

## 2. Materials and methods

### 2.1. Patients

We conducted a retrospective, single-center, descriptive study. We retrieved the medical records of JIA patients transferred from the Hacettepe University Department of Pediatric Rheumatology to the Department of Adult Rheumatology between January 2015 and May 2022. Patients with an undetermined JIA subtype and patients previously followed for JIA but who had not transitioned to adult rheumatology care at the same healthcare center were excluded. The study included 107 patients whose symptoms began before age 16 and who attended the adult rheumatology clinic for at least one follow-up visit.

### 2.2. Study design

We categorized the patients with JIA based on their pediatric diagnoses, including ERA, systemic JIA, oligoarticular JIA, RF-negative polyarticular JIA, RF-positive polyarticular JIA, and PsA. After the transition from pediatric to adult-oriented rheumatology care, 2 experienced, blinded adult rheumatologists retrospectively reclassified the patients based on the classification criteria for adults, including SpA according to the Assessment for Spondyloarthritis International Society (ASAS) criteria, rheumatoid arthritis (RA) according to the American College of Rheumatology/European League Against Rheumatism (ACR-EULAR) 2010 criteria, Still’s disease according to the Yamaguchi criteria, and PsA according to the classification criteria for psoriatic arthritis (CASPAR) during their final visits to compare with the pediatric classification [13–17]. When their classifications differed, the clinicians jointly reassessed the patients and reached a consensus on the final diagnosis. We examined the frequencies and distributions of disease groups, including radiographic axial SpA, nonradiographic axial SpA, and peripheral SpA, according to the ASAS criteria.

We obtained demographic and clinical data of the patients from medical and/or electronic hospital records. The patients’ diagnostic ages, ages at symptom onset, ages at the transition from pediatric to adult-oriented rheumatology care, durations of follow-up in both pediatric and adult rheumatology clinics, disease durations, family histories of rheumatologic conditions, comorbidities, peripheral joint and axial involvements, extra-articular manifestations (such as psoriasis, inflammatory bowel disease (IBD), uveitis), human leukocyte antigen B27 (HLA-B27), antinuclear antibody (ANA), RF, anticyclic citrullinated peptide (anti-CCP) test results, erythrocyte sedimentation rate (ESR) and C-reactive protein (CRP) values at diagnosis in the pediatric rheumatology clinic and during the final visit in the adult rheumatology clinic were meticulously documented. Additionally, we recorded the csDMARDs and bDMARDs that patients had previously used as well as those they were currently using. Radiographic sacroiliitis according to the modified New York criteria, syndesmophytes on the vertebrae, bamboo spine, hip involvement, erosions in the hands and/or feet, and sacroiliitis detected on magnetic resonance imaging (MRI) scans were also noted.

### 2.3. Statistical analysis

Statistical analysis was performed using SPSS Statistics software version 23. Visual (histograms and probability graphs) and analytical methods (Kolmogorov–Smirnov and Shapiro–Wilk tests) were used to assess the normal distribution of numerical variables. Descriptive analyses were expressed as medians (minimum–maximum) for nonnormally distributed numerical variables, and frequencies and percentages for categorical variables. The McNemar test was used to evaluate the statistical significance of the difference in proportions between 2 dependent groups. In addition, a binary logistic regression analysis with the backward stepwise method was performed to identify independent predictors of being unclassified in adulthood. Independent variables included demographic, clinical, laboratory, and imaging features. Odds ratios (ORs) with 95% confidence intervals (CIs) were calculated. Model fit was assessed using the Hosmer–Lemeshow test, and Nagelkerke R^2^ values were reported. P-values less than 0.05 were considered statistically significant.

### 2.4. Ethical considerations

We conducted the study according to the 2013 amendment of the Helsinki Declaration and obtained ethical approval from the Hacettepe University Institutional Review Board (GO: 21/734, 15 June 2021).

## 3. Results

### 3.1. Demographic features

Out of the 107 patients included in the study, 62 (57.9%) were male. The median age of the patients was 21 (17.5–27.7) years. The median age was 21 years for females (18.3–25.3) and 21 years for males (17.5–27.7). The median age at the onset of initial symptoms for the patients was 12 (0.5–15.9) years, with a median age at diagnosis of 12.6 (0.68–18.04) years. The median delay in diagnosis was 2.98 (0.33–88.94) months. The median disease duration of the patients was 9 (2–23) years. The median follow-up duration of the patients was 73.46 (0–215) months at the pediatric rheumatology clinic and 10.28 (0–68.89) months at the adult rheumatology clinic. Table 1 summarizes the demographic and clinical characteristics of the patients.

### 3.2. Pediatric and adult rheumatic disease diagnoses

ERA was identified in 53 (49.5%) patients, oligoarticular JIA in 24 (22.4%) patients, systemic JIA in 15 (14%) patients, RF-positive polyarticular JIA in 6 (5.6%) patients, RF-negative polyarticular JIA in 6 (5.6%) patients, and PsA in 3 (2.8%) patients. All patients (n = 107) were reevaluated according to the adult classification criteria with clinical, serological, and radiological findings after being transferred to adult-oriented rheumatology care. Figure illustrates the distribution of JIA subgroups and adult classifications. Table 2 summarizes the follow-up diagnoses of patients with JIA in adult-oriented rheumatology care.

A significant portion (56.6%) of the patients diagnosed with ERA were classified as having radiographic axial SpA. Among patients diagnosed with oligoarticular JIA, a notable portion (62.5%) did not meet the classification criteria used for adults. All 6 patients with RF-positive polyarticular JIA were classified as having RA. Among patients with RF-negative polyarticular JIA, half (n=3) were classified as having undifferentiated arthritis after the transition. The majority (80%) of patients initially diagnosed with systemic JIA were later diagnosed as having Still’s disease after the transition. All 3 patients diagnosed with PsA also had a diagnosis of PsA in the adult rheumatology clinic.

Multivariable logistic regression analysis showed that a history of childhood uveitis was associated with an approximately 6-fold increased risk of being unclassified in adulthood (OR = 6.3, 95% CI 1.07–36.9, p = 0.041). Other variables, including ANA positivity, disease duration, remission history, and imaging findings, were not significant predictors.

### 3.3. Laboratory findings

HLA-B27 was predominantly positive in the ERA group, with 22 out of 48 (45%) patients. RF and anti-CCP were positive in 75% of patients in the RF-positive polyarticular group. Table 2 details the serological results of the patients.

At the time of diagnosis in the pediatric rheumatology clinic, the median (minimum–maximum) values of CRP and ESR were 2.3 (0.1–29.8) and 28 (2–119), respectively. During the final visit in the adult rheumatology clinic, the median CRP and ESR values were 0.5 (0–21.6) and 9 (2–114), respectively. Both CRP and ESR levels were significantly lower during the final visit in the adult rheumatology clinic than at the time of diagnosis in the pediatric rheumatology clinic (p < 0.001).

### 3.4. Radiological findings

Radiographic assessment of the hands and/or feet during the transition showed erosion in 8 out of 46 (17%) patients. Of these patients, 4 were in the RF-positive polyarticular JIA group, 2 were in the ERA group, one was in the oligoarticular JIA group, and one was in the systemic JIA group. Radiographic sacroiliitis was recorded in 35 out of 71 (49%) patients according to the modified New York criteria. Of these patients, 30 were in the ERA group, 3 were in the oligoarticular group, and 2 were in the PsA group.

### 3.5. Extra-articular manifestations

Extra-articular involvement was present in 15 (14%) patients. The distribution of extra-articular findings is presented in Table 2. Uveitis was most common in the oligoarticular group (n = 5, 20.8%). IBD was detected only in the ERA group.

### 3.6. Comorbidities

Comorbidity was found in 40 (37.4%) patients. The most common comorbidity was familial Mediterranean fever (FMF) (n = 17, 15.8%), followed by scoliosis (n = 3, 2.8%).

### 3.7. Family history of rheumatic diseases

Eighteen (16.8%) patients had a family history of rheumatic diseases. When the patients were grouped according to the rheumatic disease diagnoses in the family history, SpA was the most common, with 8 (7.5%) patients. The family history of rheumatic diseases was most common, with 26.4% in the ERA group. Table 2 details the patients’ family history of rheumatic diseases.

### 3.8. Treatment

In the pediatric rheumatology clinic, all patients received NSAIDs as first-line therapy. The most frequently used DMARD was methotrexate (70%). Biologic agents were administered to 72 (67.3%) patients, with etanercept being the most commonly used (52.3%). During the transition, 53 (49.5%) patients were on biologic agents, 9 (8.4%) patients were on csDMARDs, and 2 (1.9%) patients were on both. Forty-three (40.2%) patients were transferred without medication. In the adult rheumatology clinic, 31 (29.9%) patients were medication-free. Forty (37.4%) patients used NSAIDs. The most frequently used csDMARD was methotrexate (12%). Biologic agents were prescribed to 57 (53.3%) patients, with etanercept being the most common (29%). Among the 43 patients who were transferred without medication, 16 required drug treatment in adult-oriented rheumatology care. Biologic agents were required by 7 out of 16 patients. In the last follow-up visit, 55 (51.4%) patients were using biologic agents. Table 3 presents a summary of the treatments administered to patients.

## 4. Discussion

In the current study, following the transition, a significant number (45.8%) of patients were classified as having axial SpA, while a notable portion (25.2%) remained unclassified. Sixty percent of the patients with JIA were under medical treatment during their transition from pediatric rheumatology to adult-oriented rheumatology care. Half of the patients were using biologic agents at their last follow-up visits in the adult rheumatology clinic.

The most prevailing subtype of JIA was ERA (49.5%), followed by oligoarticular JIA (22.4%). However, different percentages have been reported in previous studies. According to Debrach et al. [18] ERA (34.6%), oligoarticular (22.3%), and polyarticular (22.3%) JIA were the most prevalent subtypes. In another study, oligoarticular (41.6%) and polyarticular (18.7%) JIA were the most common subgroups [19]. In the Canadian JIA registry, the most commonly observed subtypes were oligoarticular (31%) and polyarticular (27%) [20]. The major variations in the reported prevalence of subtypes can be attributed to differences in study design, heterogeneity in patient populations, and the absence of transitional care programs.

After applying the adult classification criteria to each patient, 86.8% of patients in the ERA group met the ASAS criteria for either peripheral or axial involvement. This is notably higher compared to the findings of Debrach et al. [18] where 77.7% of patients met the ASAS criteria. Additionally, a significant number (83%) of patients with ERA met the diagnostic criteria for axial SpA, as corroborated by Debrach et al. [18]. ERA typically occurs with peripheral joint manifestation and enthesitis at the onset, with spinal involvement emerging later in the disease course [21]. The increased use of MRI may have improved the detection of silent and initial sacroiliac joint involvement.

In the RF-positive polyarticular JIA group, all 6 patients fulfilled the 2010 ACR/EULAR criteria, consistent with Oliveira-Ramos et al. [22], supporting the view that RF-positive polyarticular JIA corresponds to adult seropositive RA [23]. Likewise, all three patients with psoriatic JIA met the CASPAR criteria, in agreement with Debrach et al. [18]. Our findings, however, should be interpreted with caution, given the small sample size.

Among the 6 patients diagnosed with RF-negative polyarticular JIA, 33.3% fulfilled the 2010 ACR/EULAR criteria for RA. It is noteworthy that half of the patients within this subgroup failed to meet any of the established criteria for adult rheumatologic diseases.

Within the oligoarticular group, most patients (62.5%) were categorized as unclassified according to the adult classification criteria, as indicated by Debrach et al. [18]. These data reinforce the previous claim that oligoarticular JIA presents unique characteristics specific to the pediatric population [23–25]. The oligoarticular JIA has no direct adult counterpart, making it one of the most challenging subtypes to manage. This further underscores the limited validity of applying adult classification criteria to childhood-onset disease and highlights the need for JIA-specific classification frameworks for the transitional phase.

A majority (80%) of the patients in the systemic JIA group satisfied the Yamaguchi criteria for Still’s disease. Debrach et al. [18] reported a slightly lower prevalence at 66.6%. Systemic JIA and adult-onset Still’s disease are now considered to be the same disease with different onset ages, and both are referred to as Still’s disease.

Multivariable logistic regression analysis identified that a history of childhood uveitis was the only statistically significant predictor of being unclassified in adulthood, with an approximately 6-fold increase in likelihood. To our knowledge, no comparable data have previously been reported, so we consider that this finding may contribute to the existing literature.

The rate of uveitis in JIA varies widely in the literature, ranging from 4% to 24% [26, 27]. This variability depends on the population studied and geographic location. Additionally, oligoarticular subtype, early onset arthritis, and ANA positivity have been shown as risk factors for the development of uveitis in patients with JIA [26, 27]. In the current study, the prevalence of uveitis was 8.4% among all JIA patients. This supports previous studies, especially in the oligoarticular group at 20.8%. Debrach et al. [18] identified uveitis in 17 out of 130 (13.1%) patients in all JIA patients and 9 out of 29 (31%) patients in the oligoarticular subgroup. In another study, uveitis was found in 12% of all JIA patients, especially in an oligoarticular subgroup at 41% [28]. We found that 57.1% of patients with uveitis were ANA positive. However, within the oligoarticular subgroup, 75% of patients with uveitis were ANA positive.

HLA-B27 positivity has been shown as a risk factor for the development of uveitis in patients with ERA [29]. In our study, uveitis was observed in 3 out of 53 (5.7%) patients with ERA, and all affected patients were HLA-B27 positive. In 2 previous studies, uveitis was reported in 15.6% and 7% of patients in the ERA group. The majority of these patients were HLA-B27 positive, with rates of 100% and 75%, respectively [18, 28].

ERA and familial autoimmune disease history are predictive factors for the development of IBD in patients with JIA [30]. Furthermore, fecal calprotectin levels were elevated in patients with ERA compared to healthy controls, patients with connective tissue disease, and the other JIA subgroups [31]. Our findings indicated that 2.8% of the patients with JIA presented with IBD; notably, all affected patients were within the ERA subgroup. Another study reported Crohn’s disease in 2.2% of patients diagnosed with ERA [18]. Consequently, when gastrointestinal symptoms manifest in a patient with ERA, it is essential to conduct assessments aimed at ruling out the presence of IBD [32].

The most concomitant disease in our cohort was FMF, observed in 15.8% of patients. In a multicenter study conducted in Türkiye, FMF was reported in 3.3% of patients with JIA [33]. Moreover, concomitant FMF negatively influences the clinical course of JIA, highlighting the potential impact of region-specific comorbidities on disease phenotype [34]. Although establishing a clear mechanistic relationship between FMF and JIA remains challenging, clinicians should consider FMF in patients of Mediterranean origin with JIA who present with recurrent extra-articular symptoms. Early recognition of such comorbidities may provide important clues about disease presentation and support more tailored management strategies in this regional context.

In our study, 64 (60%) patients received medical treatment during transition, with 49.5% receiving biologic agents. While certain biologic therapy courses may have been terminated, the utilization of biologic agents during patient follow-up at the adult rheumatology unit modestly increased to 51.4%. Vidqvist et al. [35] showed that 23% of patients were on biologic therapy during the transition, and the use of biologics increased to 29% during adult care. In the Canadian registry, 96 out of 131 (73%) patients were on medication, with 31% receiving biologic agents during transition [20]. The literature did not corroborate our results; the discrepancies may stem from the increased adoption of biological treatment methods over time, variances in healthcare systems, and/or differences in access to biologic therapies.

There are several limitations of this study. The number of patients included in the study was low, and the follow-up period at the adult rheumatology unit was short (median 10.28 months). Additionally, due to the retrospective design of the study, an assessment of the patients’ disease activity could not be performed. Our study was conducted in a tertiary center that may have led to a selection bias toward more severe cases. This should be taken into account when interpreting our findings. Nevertheless, our study provides an overview of contemporary treatment approaches for patients with JIA during their transition to adult-oriented rheumatology care.

In conclusion, a significant number of patients in oligoarticular and RF-negative polyarticular JIA groups did not meet adult classification criteria, making them the most challenging subtypes to manage in adult-oriented rheumatology care. The implementation of continuous transitional care, alongside the development of JIA-specific classification frameworks tailored to the transitional phase, is essential for improving the prognosis of JIA. Prospective longitudinal studies with long follow-up periods would be invaluable for investigating the natural history of JIA in adulthood.

## Figures and Tables

**Figure:**
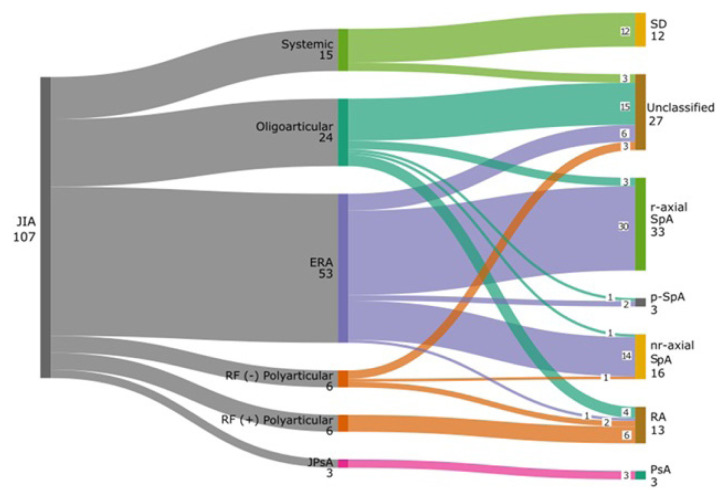
The distribution of JIA subgroups and adult classifications. ERA, enthesitis-related arthritis; JIA, juvenile idiopathic arthritis; JPsA, juvenile psoriatic arthritis; nr-axial SpA, nonradiographic axial spondyloarthritis; PsA, psoriatic arthritis; p-SpA, peripheral spondyloarthritis; RA, rheumatoid arthritis; r-axial SpA, radiographic axial spondyloarthritis; RF, rheumatoid factor; SD, Still’s disease.

**Table 1:** The characteristics of the patients with JIA (n = 107).

Characteristics	n (%) or median (min-max)

Male	62 (57.9)

Age at symptom onset, years	11.98 (0.55–15.9)

Age at diagnosis, years	12.61 (0.68–18.04)

The time between the onset of symptoms and the diagnosis, months	2.98 (0.33–88.94)

Disease duration, years	9 (2–23)

Diagnosis	
Enthesitis-related arthritis	53 (49.5)
Oligoarticular JIA	24 (22.4)
Systemic JIA	15 (14)
RF-positive polyarticular JIA	6 (5.6)
RF-negative polyarticular JIA	6 (5.6)
Psoriatic arthritis	3 (2.8)

Comorbidities	40 (37.4)

Family history of rheumatic disease	18 (16.8)

Duration of follow-up in pediatric rheumatology, months	73.46 (0–215)

Time between last pediatric examination and first adult examination, months	3.70 (0–73.12)

Age at first adult rheumatology examination, years	19.54 (16.48–23.33)

Age at last adult rheumatology examination, years	20.94 (17.53–27.73)

Remission at the time of transition	78 (72.9)

Duration of follow-up in adult rheumatology, months	10.28 (0–68.89)

JIA, Juvenile idiopathic arthritis; RF, rheumatoid factor.

**Table 2:** Classification of JIA within the spectrum of adult rheumatic diseases.

		Oligoarticular JIA (n = 24)	Polyarticular JIA, RF+ (n = 6)	Polyarticular JIA, RF− (n = 6)	ERA (n = 53)	PsA (n = 3)	Systemic JIA (n=15)	Total (n = 107)
**Sex ratio (F/M)**		14/10	5/1	4/2	12/41	1/2	9/6	45/62
**Adult classifications**	**r-axial SpA**	3 (12.5)	0	0	30 (56.6)	0	0	33 (30.8)
	**Nr-axial SpA**	1 (4.2)	0	1 (16.7)	14 (26.4)	0	0	16 (15)
	**p-SpA**	1 (4.2)	0	0	2 (3.8)	0	0	3 (2.8)
	**RA**	4 (16.7)	6 (100)	2 (33.3)	1 (1.9)	0	0	13 (12.1)
	**PsA**	0	0	0	0	3 (100)	0	3 (2.8)
	**SD**	0	0	0	0	0	12 (80)	12 (11.2)
	**Unclassified**	15 (62.5)	0	3 (50)	6 (11.3)	0	3 (20)	27 (25.2)
**Serology**	**HLA-B27**	3/17 (17)	0/3 (0)	1/3 (33)	22/48 (45)	1/2 (50)	1/4 (25)	28/77 (36)
	**RF**	0/16 (0)	3/4 (75)	1/5 (20)	0/22	0/1 (0)	0/9 (0)	4/57 (7)
	**Anti-CCP**	0/15 (0)	3/4 (75)	0/1 (0)	0/15	0/1 (0)	0/7 (0)	3/43 (6)
	**ANA**	13/22 (59)	4/5 (80)	2/6 (33)	14/26 (53)	1/1 (100)	3/10 (30)	37/70 (52)
**Structural damage**	**Erosions**	1/11 (9)	4/6 (66)	0/2 (0)	2/19 (10)	0/2 (0)	1/6 (16)	8/46 (17)
	**Sacroiliitis** *	3/13 (23)	0/2 (0)	0/4 (0)	30/46 (65)	2/3 (66)	0/3 (0)	35/71 (49)
**Extra-articular manifestations**	**Uveitis**	5 (20.8)	0	1 (16.7)	3 (5.7)	0	0	9 (8.4)
	**Psoriasis**	0	0	0	0	3 (100)	0	3 (2.8)
	**IBD**	0	0	0	3 (5.7)	0	0	3 (2.8)
**Family history of rheumatic disease**	**SpA**	0	0	0	8 (15)	0	0	8 (7.5)
	**RA**	1 (4.2)	1 (16.7)	0	2 (3.8)	0	1 (6.7)	5 (4.7)
	**FMF**	0	0	0	2 (3.8)	1 (33.3)	0	3 (2.8)
	**IBD**	0	0	0	1 (1.9)	0	0	1 (0.9)
	**BD**	0	0	0	1 (1.9)	0	0	1 (0.9)

Data depicted as numbers (%). ANA, antinuclear antibody; Anti-CCP, Anticyclic citrullinated peptide antibody; BD, Behcet’s disease; ERA, enthesitis-related arthritis; F, female; FMF, familial Mediterranean fever; HLA, human leukocyte antigen; IBD, inflammatory bowel disease; JIA, Juvenile idiopathic arthritis; M, male; nr-axial SpA, nonradiographic axial spondyloarthritis; PsA, psoriatic arthritis; p-SpA, peripheral spondyloarthritis; RA, rheumatoid arthritis; r-axial SpA, radiographic axial spondyloarthritis; RF, rheumatoid factor; SD, Still’s disease.

* According to the modified New York Criteria

**Table 3:** Treatments used in patients with JIA in pediatric and adult rheumatology units.

Treatment, n (%)	Pediatric Rheumatology	Adult Rheumatology	P-value

NSAID	107 (100)	75 (70.1)	**<0.001**

Methotrexate	75 (70.1)	13 (12.1)	**<0.001**

Sulfasalazine	36 (33.6)	5 (4.7)	**<0.001**

Leflunomide	0 (0)	2 (1.9)	0.50

Hydroxychloroquine	1 (0.9)	6 (5.6)	0.12

Cyclosporine	4 (3.7)	0 (0)	0.12

Systemic corticosteroids	37 (34.6)	13 (12.1)	**<0.001**

Biologic agents:	72 (67.3)	57 (53.3)	**0.001**
Etanercept	56 (52.3)	31 (29)	**<0.001**
Adalimumab	18 (16.8)	11 (10.3)	0.11
Infliximab	5 (4.7)	2 (1.9)	0.37
Certolizumab	0 (0)	3 (2.8)	0.25
Secukinumab	2 (1.9)	1 (0.9)	1.00
Anakinra	11 (10.3)	1 (0.9)	**0.002**
Canakinumab	5 (4.7)	3 (2.8)	0.62
Tocilizumab	8 (7.5)	8 (7.5)	1.00
Janus kinase inhibitor	0 (0)	2 (1.9)	0.50

NSAID, nonsteroidal anti-inflammatory drug.

* p < 0.05 indicates a statistically significant difference from the adult rheumatology.
